# Meta-analysis of efficacy and safety of intravenous ferric carboxymaltose (Ferinject) from clinical trial reports and published trial data

**DOI:** 10.1186/1471-2326-11-4

**Published:** 2011-09-24

**Authors:** R Andrew Moore, Helen Gaskell, Peter Rose, Jonathan Allan

**Affiliations:** 1 Pain Research, Nuffield Department of Anaesthetics, University of Oxford, Oxford Radcliffe Hospitals, The Churchill, Oxford, OX3 7LJ, UK; 2 Department of Clinical Gerontology, John Radcliffe Hospital, Oxford, OX3 9DU, UK; 3 Haematology Department, Warwick Hospital, Lakin Road, Warwick, CV35 5BW, UK; 4 Pharmacy Department, Beatson West of Scotland Cancer Centre, Glasgow, G12 0YN, UK

## Abstract

**Background:**

Recommendations given for intravenous iron treatment are typically not supported by a high level of evidence. This meta-analysis addressed this by summarising the available date from clinical trials of ferric carboxymaltose using clinical trial reports and published reports.

**Methods:**

Clinical trial reports were supplemented by electronic literature searches comparing ferric carboxymaltose with active comparators or placebo. Various outcomes were sought for efficacy (attainment of normal haemoglobin (Hb), increase of Hb by a defined amount, for example), together with measures of harm, including serious adverse events and deaths.

**Results:**

Fourteen studies were identified with 2,348 randomised patients exposed to ferric carboxymaltose, 832 to oral iron, 762 to placebo, and 384 to intravenous iron sucrose. Additional data were available from cohort studies. Intravenous ferric carboxymaltose was given up to the calculated iron deficit (up to 1,000 mg in one week) for iron deficiency anaemia secondary to chronic kidney disease, blood loss in obstetric and gynaecological conditions, gastrointestinal disease, and other conditions like heart failure. The most common comparator was oral iron, and trials lasted 1 to 24 weeks. Intravenous ferric carboxymaltose improved mean Hb, serum ferritin, and transferrin saturation levels; the mean end-of-trial increase over oral iron was, for Hb 4.8 (95% confidence interval 3.3 to 6.3) g/L, for ferritin 163 (153 to 173) μg/L, and for transferrin saturation 5.3% (3.7 to 6.8%). Ferric carboxymaltose was significantly better than comparator in achievement of target Hb increase (number needed to treat (NNT) 6.8; 5.3 to 9.7) and target Hb NNT (5.9; 4.7 to 8.1). Serious adverse events and deaths were similar in incidence in ferric carboxymaltose and comparators; rates of constipation, diarrhoea, and nausea or vomiting were lower than with oral iron.

**Conclusions:**

This review examined the available trials of intravenous ferric carboxymaltose using details from published papers and unpublished clinical trial reports. It increases the evidence available to support recommendations given for intravenous iron treatment, but there are limited trial data comparing different intravenous iron preparations.

## Background

Anaemia is common. A 2008 WHO report concentrating on pre-school children and women estimated that worldwide one in four persons is affected by anaemia, with pregnant women and preschool-age children at the greatest risk [1]. High prevalence of anaemia is associated with older age [2], and with acute and chronic conditions, like chronic kidney disease [3].

Blood is expensive. In 2000/2001 the estimated UK NHS cost for an adult transfusion was £635 for red blood cells (RBC), £378 for fresh frozen plasma, £347 for platelets, £834 for cryoprecipitate, with large increases over the preceding decade [4]. The cost of providing red blood cell transfusions to surgical patients in the USA, including major process steps, staff, consumables, and direct and indirect overhead costs amounted to between US$522 and US$1183 [5]. The UK Blood Transfusion Service puts the cost of providing one unit of RBC at about £130 [6], but the total annual cost of provision and transfusion of blood products was put at £898 millions in 2000/1, with an annual increase of about 17% [4].

Despite differences in time and situation, it amounts to an expensive business; it is sensible to avoid these costs and the unnecessary use of blood where possible. There are also potential risks, of incompatibility, infection, and iron overload in patients having recurrent transfusions over long periods.

For iron deficiency anaemia associated with conditions like chronic kidney disease (CKD) the first recourse is to iron supplementation, either oral or parenteral. Anaemia is associated with increased morbidity, and with increased mortality in chronic conditions [7-12], though high Hb levels may be harmful [13,14]. Anaemia is also associated with huge economic burden [15].

For CKD, for example, iron deficiency is a frequent cause of anaemia, resulting from multiple blood sampling, interventional procedures, gastrointestinal bleeding and poor nutritional intake [3]. The severity of iron deficiency anaemia increases with advancing chronic kidney disease, particularly in patients on haemodialysis [16]. The combination of CKD and a low Hb level occurs in about 100,000 people in the UK [3]. Maintenance of target range Hb levels (11-12 g/dL) in non-dialysis-dependent patients with CKD is associated with positive patient outcomes and improved quality of life and physical function [16]. The complex interrelationships between anaemia and cardiorenal function currently being teased out indicate that we have much to learn [17].

Treatment of iron deficiency anaemia means identifying and treating its cause, and replacing iron may be only part of that [18,19]. Oral iron is neither suitable nor effective in all patients; oral iron is poorly absorbed, and is not well tolerated because of adverse gastrointestinal effects [20-22]. Intravenous iron preparations were developed to overcome these problems in patients in whom oral iron is poorly tolerated, or more rapid replacement is required, or absorption is compromised. Intravenous iron preparations have included iron as high or low molecular weight iron dextran, iron gluconate, or iron sucrose, and ferric carboxymaltose; differences include the number of administrations required to replenish iron stores. Some, notably iron dextrans with higher molecular weight, have been associated with hypersensitivity reactions that have limited their use [23,24].

In some conditions, notably CKD, other factors are important, particularly the appropriate balance between stimulation of erythropoiesis and the provision of iron for the manufacture of Hb [3], and the routine use of erythropoiesis-stimulating agents (ESAs) has led to a need for concomitant iron supplementation. Together, ESAs and iron now form the cornerstone of anaemia management in chronic kidney disease. Intravenous iron supplementation is effective in CKD, with acceptable safety; it permits replacement of iron stores for erythropoiesis, and improves the responsive to ESAs, as well as reducing the requirement for costly ESA therapy and transfusions [22,25-27].

Iron sucrose is typically administered as a slow push injection or a 15- to 30-minute infusion in doses of 100-200 mg, requiring multiple outpatient visits and repeated intravenous access for patients to receive the standard therapeutic course of 1,000 mg elemental iron. Iron dextran can be administered as a single dose, but this requires administration over a period of four to six hours. In addition, iron dextran complexes can cause fatal dextran-induced anaphylactic reactions [3,28]. Anaphylactic reaction is rare, with estimates for iron dextran products of 0.6% incidence [29].

Ferric carboxymaltose (Ferinject^®^) is a novel non-dextran-containing complex of iron that allows for administration of a large replenishment dose (≤1,000 mg of iron) over a short infusion period (15-30 minutes), typically to the amount required for iron repletion. Ferric carboxymaltose is effective in improving Hb concentrations in non-dialysis-dependent patients with CKD [30]. It may be of significant benefit for use in the outpatient department or in a community setting as a result of its rapid and high-dose replacement of depleted iron stores in patients with CKD, as well as in various other adult populations with iron deficiency anaemia [30].

It has been noted that many of the recommendations for intravenous iron treatment are not supported by a high level of evidence [31]. This meta-analysis seeks to address the evidence gap by summarising the available data from clinical trials of ferric carboxymaltose using clinical trial reports and published results. Clinical trial reports frequently contain much more detail than is available in published reports, if only because they are not restricted by word count limits; meta-analyses from clinical trial reports of other drugs have been conducted previously [32-34].

## Methods

### Identification of studies

We sought randomised trials and cohort studies of the use of intravenous ferric carboxymaltose for the treatment of iron deficiency anaemia. Studies were included if they treated iron deficiency anaemia, of any cause, for any duration, in patients of any age, and included at least 10 patients. Case reports or informal case series were not eligible.

Vifor Pharma UK Limited provided PDF versions of CTRs of phase 2/3 studies of IV ferric carboxymaltose (Ferinject^®^), irrespective of clinical condition or design. Published studies were identified by searching PubMED, using as key words Ferinject, ferric carboxymaltose, or iron carboxymaltose in title, abstract, or anywhere in a document. Articles identified were examined online to assess whether they might be a clinical study of efficacy or harm, and hard copy obtained of any regarded as probably being randomised trials or cohort studies. In addition, references of retrieved articles, CTRs, and review articles [30,35,36] were examined for any otherwise unidentified studies.

Electronic searches were conducted originally in June 2010, with additional searches at the time of submission in February 2011. CTRs from Vifor Pharma were of trials with completed reports up to March 2010.

### Outcomes sought

We sought various different outcomes from studies. For efficacy we sought outcomes approximating the following:

• Treatment success, however defined (increase in Hb by a defined amount, increase in Hb above a defined level, increase in Hb plus increases in ferritin and transferrin saturation (TSAT)).

• Attainment of normal Hb, however defined.

• Increase of Hb by a defined amount.

• Mean increase in Hb at various times after start of treatment.

• Mean increase in serum ferritin at various times after start of treatment.

• Mean increase in TSAT at various times after start of treatment.

For measures of harm, we sought the following:

• Patients experiencing at least one adverse event.

• Patients experiencing at least one serious adverse event.

• Patients dying.

• Patients experiencing hypertension.

• Patients withdrawing for any reason, because of adverse events, or because of evidence of lack of efficacy (transfusion, introduction of or change in EPO dose, for example).

• Patients experiencing adverse events, as defined by organs or body area.

• Patients with a specific adverse event like constipation or headache.

We also sought any information about change of haematological parameters of Hb, serum ferritin, and TSAT over time.

### Analysis

Data were extracted from studies by one author, and independently verified by another author. Any disagreement was resolved by consensus, and with the aid of another experienced researcher not connected with the review.

Studies were assessed for quality using the Oxford Quality Scale [37]. We defined intention to treat as a patient being randomised and receiving at least one dose of any treatment, and used this as the denominator where possible.

Analyses were planned in the following way:

1. The type of comparator would determine the primary analysis (placebo, oral iron, other IV iron preparation, blood). While combined analysis with different comparators was planned, the main focus would be with the particular comparators for efficacy.

2. Any heterogeneity between clinical conditions would be explored using L'Abbé plots [38], and subgroup analyses if appropriate.

Dichotomous and continuous data were entered into RevMan 5.0 using the appropriate statistic. For dichotomous outcomes, dichotomous data were used to calculate relative benefit or risk (RB, RR) with 95% confidence intervals (CIs) using a fixed-effect model [39]. The number needed to treat to benefit (NNT) was calculated as the reciprocal of the absolute risk reduction [40]. For unwanted events, the NNT becomes the number needed to treat to harm (NNH), or the number needed to treat to prevent harm (NNTp).

For mean change over time, we planned, for each parameter, to calculate a weighted mean by dividing the sum of the product of mean change in concentration and number of patients of individual studies by the sum of the number of patients in all studies combined.

For all calculations the intention was to pool data for analysis only when available from at least two trials and with data available from at least 400 patients.

## Results

### Available studies

Electronic searches using PubMED produced 33 possible titles; of these eight reports of randomised trials and one report of a cohort study were included (Additional file 1; flow diagram). Vifor provided eleven CTRs. Some published papers duplicated CTRs, some [41-43] did not, and some CTRs contained data that were not published. Identification of possible duplications was achieved by matching condition, numbers enrolled and treated, and patient characteristics between CTRs and published studies. One large randomised trial became available after submission, and was included at revision, before resubmission. Of the 11 randomised trials, five scored 5/5, seven scored 3/5 (because they were open studies) and one 2/5; scores of 3 or more out of 5 on this scale are associated with a low potential for bias.

Table 1 shows the sources of information for the available studies and the relationship between CTRs and published studies. Information regarding efficacy or harm was taken from sources that reported them. We finally included 14 unique studies, 11 randomised trials and three cohort studies:

**Table 1:** Available studies (NCT = ClinicalTrials.gov registry number)

CTR	Condition	NCT	Published study
1VIT04004 [44]	Chronic kidney disease		Qunibi et al. Nephrol Dial Transplant 2010 doi: 10.1093/ndt/gfq613 [45]
1VIT04005 [47]	Chronic kidney disease		No publication identified
VIT-IV-CL-015 [46]	Haemodialysis		No publication identified
53214 [48]	Haemodialysis		Covic & Mircescu. Nephrol Dial Transplant 2010 25: 2722-2730 [49]
VIT-IV-CL-009 [50]	Post partum anaemia		Breymann et al. Int J Gynecol Obstet 2008 101: 67-73 [51]
1VIT06011 [52]	Post partum anaemia		Seid et al. Am J Obstet Gynecol 2008 199:435.e1-7 [53]
1VIT04002/4003 [54]	Uterine bleeding	NCT00395993	Van Wyck et al. Transfusion 2009 49: 2719-2728 [41]
No CTR available	Post partum anaemia	NCT00396292	Van Wyck et al. Obst & Gynecol 2007 110:267-278 [55]
VIT-IV-CL-03 [56]	GI causes of anaemia		No publication identified
VIT-IV-CL-008 [57]	Inflammatory bowel disease		Kulnigg et al. Am J Gastroeneterol 2008 24:1507-1523 [58]
1VIT05006 [59]	Iron deficiency anaemia		Bailie et al. Hemodialysis International 2010 14: 47-54 [35]
CARS 1 [60]	Iron deficiency anaemia		No publication identified
No CTR available	Congestive heart failure	NCT00520780	Anker et al. NEJM 2009 361 [42]
No CTR available	CKD design	NCT00981045	Szczech et al. Nephrol Dial Transplant 2010 25: 2368-2375 [61]

• In iron deficiency anaemia secondary to kidney disease, four studies provided data. Two were randomised, open studies comparing IV ferric carboxymaltose with oral iron [44,45] or IV Venofer (iron sucrose [46]). Two were cohort studies [47-49].

• In iron deficiency anaemia secondary to blood loss during childbirth or associated with heavy uterine bleeding, four randomised, open studies provided data [41,50-55]. All compared IV ferric carboxymaltose with oral iron.

• In iron deficiency anaemia secondary to gastrointestinal causes, one cohort study [56] and one randomised open study [57,58] provided data comparing IV ferric carboxymaltose with oral iron. One randomised trial compared IV ferric carboxymaltose with IV iron sucrose [43].

• Three randomised, double blind studies compared IV ferric carboxymaltose with placebo in different conditions. 1VIT05006 [35,59] was a one-week crossover and CARS-1 [60] was a placebo and active controlled study over 12 weeks in patients with iron deficiency anaemia secondary to several different conditions. In congestive heart failure, IV ferric carboxymaltose was compared with placebo over 24 weeks [42].

• One report of a trial design in patients with iron deficiency anaemia in the setting of non-dialysis-dependent chronic kidney disease [61] had no results yet available.

The 14 studies varied in duration between one and 24 weeks and involved 3933 patients, some of whom were exposed to treatments more than once because of crossovers or re-randomisation into extension studies. Therefore 2,348 patients were exposed to IV ferric carboxymaltose in randomised trials and 348 in cohort studies. Oral iron was administered to 832 patients, iron sucrose to 384, and placebo to 762 in randomised trials. Typically, IV ferric carboxymaltose was given up to the calculated iron deficit, with no more than 1,000 mg iron administered in a single week; thereafter smaller doses were given on a regular basis, in, for instance, chronic kidney disease. Details of the study designs, patients participating, and outcomes, are given in Additional file 2 (Trial details).

### Efficacy analyses - comparison with oral iron

#### 1 Mean change in values over time

Five studies comparing IV ferric carboxymaltose with oral iron provided data to allow a calculation of the weighted mean change over time in Hb, serum ferritin, and TSAT [44,50,52,54,57]. At least three studies had to provide data for a weighted mean to be calculated at any time period; because studies had different duration (typically up to 12 weeks for randomised studies), calculation of the weighted mean over weeks 6-12 was done with the value available at the longest duration. For ferric carboxymaltose, between 536 and 791 patients contributed data, and for oral iron between 439 and 598 contributed. Estimates of statistical differences were not possible due to differing contributions from studies at different time points, and inconsistent reporting of dispersion.

**Figure 1:**
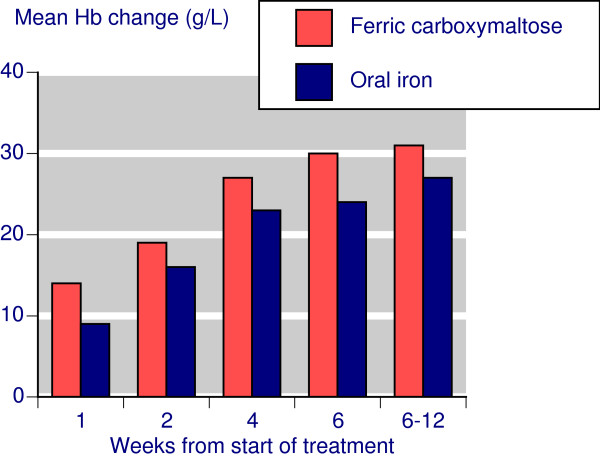
**Time course for Hb changes**.

Following treatment with IV ferric carboxymaltose, the mean Hb change from baseline increased steadily over weeks 1 to 6, with a mean increase of about 30 g/L by week 6 and sustained at that level to week 12 (Figure 1). With oral iron somewhat smaller increases followed a similar time course.

**Figure 2:**
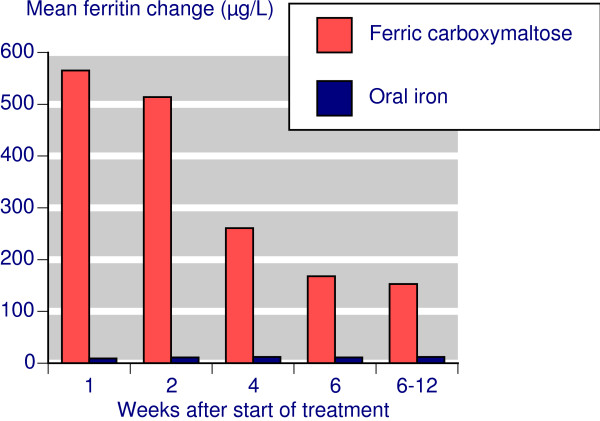
**Time course for serum ferritin changes**.

Following treatment with IV ferric carboxymaltose, the serum ferritin change from baseline declined steadily over weeks 1 to 6, with a mean increase of about 150 μg/L by week 6 sustained at that level to week 12 (Figure 2). With oral iron only small changes in serum ferritin occurred.

**Figure 3:**
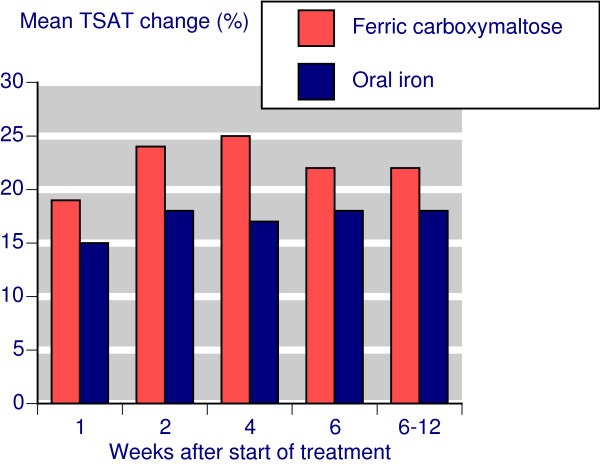
**Time course for TSAT changes**.

Following treatment with IV ferric carboxymaltose, the mean TSAT increased steadily from baseline to week 4 and then declined slightly; the mean increase was about 22% by week 6 and remained at that level at that level to week 12 (Figure 3). With oral iron somewhat smaller increases were seen.

#### 2 Hb Responders

**Figure 4:**
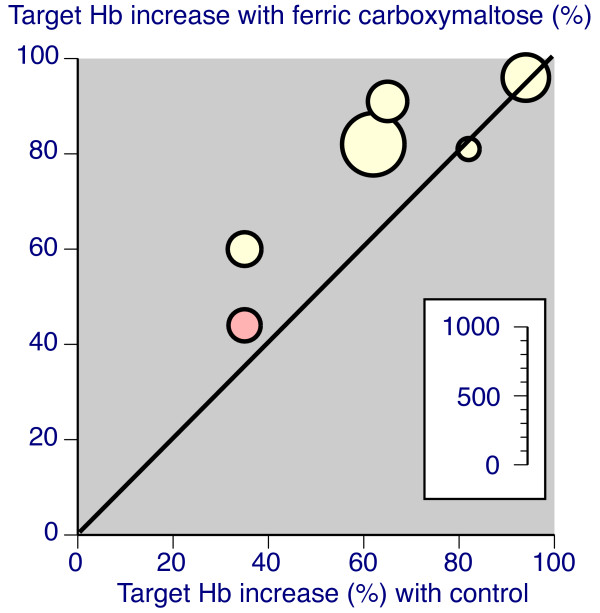
**L'Abbé plots for percentage of patients achieving a target Hb increase (yellow with oral iron comparator, red for intravenous iron sucrose (Venofer))**. Each symbol represents one trial, with size of symbol proportional to number of patients randomised using the inset scale.

**Figure 5:**
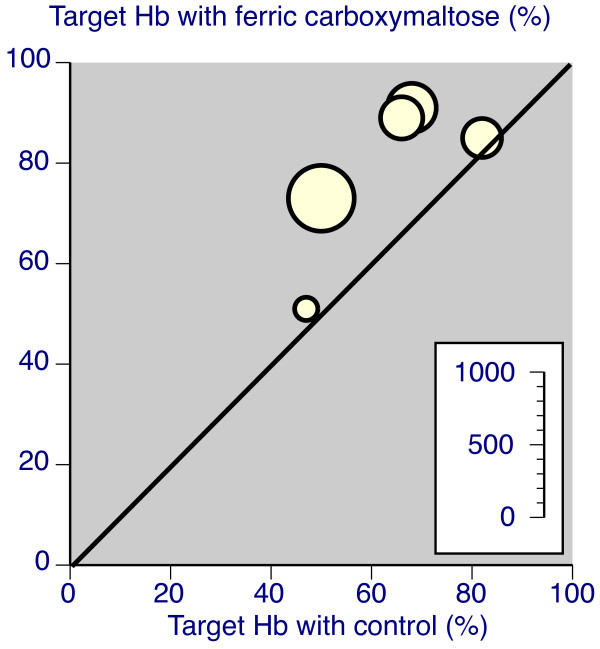
**L'Abbé plots for percentage of patients achieving a target Hb (yellow with oral iron comparator)**. Each symbol represents one trial, with size of symbol proportional to number of patients randomised using the inset scale.

**Figure 6:**
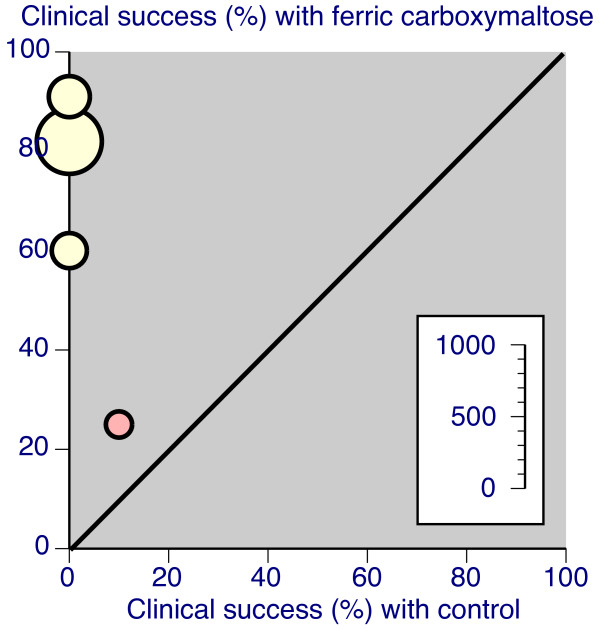
**L'Abbé plots for percentage of patients achieving clinical success, however defined (yellow with oral iron comparator, red for intravenous iron sucrose (Venofer))**. Each symbol represents one trial, with size of symbol proportional to number of patients randomised using the inset scale.

Response was defined in various ways: by achieving a target Hb increase (typically ≥20 g/L increase), by achieving a target Hb level (typically ≥120 g/L), or by achieving what was typically described as clinical success in the original reports (typically a rise in Hb of ≥20 g/L plus ferritin increase of ≥150 μg/L or TSAT increase of ≥20%). Some studies used several of these definitions. Table 2 shows the results of analyses for responders, for all comparators, and for oral iron only. There was good consistency between trial results for all three definitions of responder, with studies demonstrating considerable homogeneity, as the L'Abbé plots in Figures 4, 5, and 6 show.

**Table 2:** Hb responder analysis

		Number of		Percent with			
				
Outcome	Comparator	Trials	Patients	Ferric carboxymaltose	Control	RB 95% CI	NNT 95% CI
Target Hb increase	All	6	1715	78	63	1.2 (1.2 to 1.3)	6.8 (5.3 to 9.7)
	Oral iron	5	1481	74	59	1.3 (1.2 to 1.4)	6.6 (5.0 to 9.6)

Achieve target Hb level	Oral iron	5	1509	79	62	1.3 (1.2 to 1.4)	5.9 (4.7 to 8.1)

Clinical success	All	4	1167	70	2	37 (20 to 67)	1.5 (1.4 to 1.6)
	Oral iron	3	984	78	0	250 (51 to 1190)	1.3 (1.2 to 1.3)

**Figure 7:**
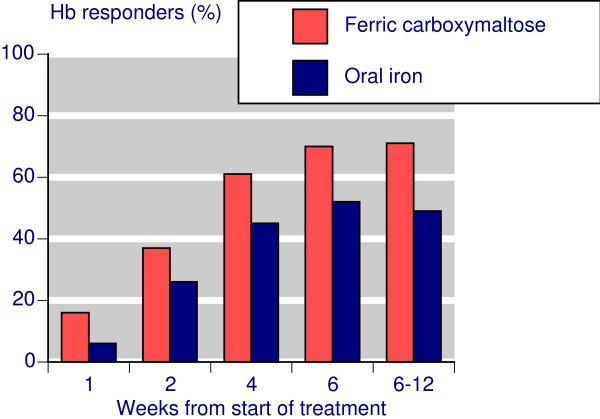
**Time course of Hb responders**.

With IV ferric carboxymaltose 70-80% of patients achieved target Hb increases or target Hb levels, somewhat more than with controls, and producing NNTs of about 6 compared with oral iron. For clinical success similar high levels of response were found for IV ferric carboxymaltose, but the failure of oral iron in particular to increase ferritin levels resulted in low rates of clinical success, with consequent high relative benefit, and low (good) NNTs of about 1.5. The time course of response (Figure 7) showed that maximum response rates were typically achieved by 4-6 weeks.

#### 3 Serum ferritin and TSAT responders

Responder definitions were also used for serum ferritin and TSAT in three studies [46,50,57]. For ferritin the definition was for serum ferritin at four weeks to be between a minimum of 50, 100, or 200 μg/L and a maximum of 800 μg/L. This was achieved by about 60% after IV ferric carboxymaltose, but lower numbers of controls. In comparison with oral iron, the NNT to achieve target ferritin at four weeks was about 3 (Table 3). For TSAT, about 65% achieved target values of being in the range of 20-50% at four weeks with IV ferric carboxymaltose, about the same as with control (Table 3).

**Table 3:** Serum ferritin and TSAT responder analysis

		Number of		Percent with			
				
Outcome	Comparator	Trials	Patients	Ferric carboxymaltose	Control	RB 95% CI	NNT 95% CI
Target ferritin increase	All	3	611	61	43	1.6 (1.3 to 1.9)	5.6 (3.9 to 10)
	Oral iron	2	428	59	22	2.7 (2.0 to 3.6)	2.7 (2.2 to 3.6)

Target TSAT increase	All	3	611	65	62	1.1 (0.9 to 1.2)	not calculated
	Oral iron	2	428	64	59	1.1 (0.9 to 1.3)	not calculated

#### 4 Mean changes

**Figure 8:**
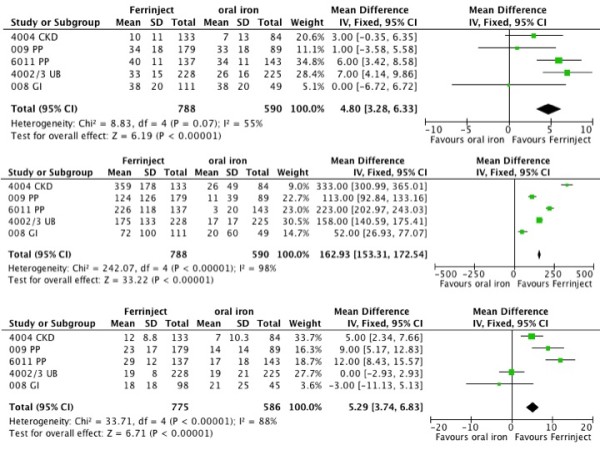
**Mean improvements with intravenous ferric carboxymaltose and oral iron, for Hb (8a), ferritin (8b), and TSAT (8c)**.

Studies also reported mean changes in Hb, serum ferritin, and TSAT over the duration of the study. Figure 8 shows the difference between IV ferric carboxymaltose and oral iron in five trials reporting data. For Hb, the mean improvement over oral iron was 4.8 (95%CI 3.3 to 6.3) g/L. For serum ferritin, the mean improvement was 163 (95%CI 153 to 173) μg/L. For TSAT, the mean improvement was 5.3 (95%CI 3.7 to 6.8)%. All were statistically significant improvements over oral iron.

### Efficacy analyses - active comparisons and cohort studies

One randomised double blind placebo controlled trial provided information on efficacy of IV ferric carboxymaltose in anaemia associated with heart failure. Another randomised open study compared IV ferric carboxymaltose with IV iron sucrose. Three cohort studies also provided additional information on the use of ferric carboxymaltose.

#### Active comparisons

A randomised double blind trial of IV ferric carboxymaltose for treating heart failure randomised 459 patients to IV ferric carboxymaltose or IV placebo, and reported results at 24 weeks, according to initial Hb level [42]. Half the patients had Hb levels ≤120 g/L initially, and for these mean final Hb was higher (127 g/L) after IV ferric carboxymaltose than IV placebo (118 g/L). Final levels of ferritin (275 vs 68 μg/L) and TSAT (29% vs 17%) were also higher after IV ferric carboxymaltose than placebo.

A randomised open trial compared IV ferric carboxymaltose with IV iron sucrose for treating iron deficiency anaemia secondary to inflammatory bowel disease (ulcerative colitis and Crohn's disease) in 475 patients [43]. Patients had initial Hb levels below 120 g/L in women or 130 g/L in men. The primary outcome of increase in Hb by at least 20 g/L was achieved by 66% with ferric carboxymaltose and 54% with iron sucrose. Achievement of a normal Hb occurred in 73% with ferric carboxymaltose and 62% for iron sucrose; normal TSAT (20-50%) was achieved in 53% and 36%, and normal ferritin (≥100 μg/L) by 43% and 27% respectively. The time course for these changes was similar to that seen in the oral iron comparisons.

This study also compared cost effectiveness of the two regimens. Although ferric carboxymaltose has a higher cost per treatment than iron sucrose (US$ 311 vs 154), the greater number of infusions with iron sucrose resulted in a higher overall treatment cost (US$ 653 for ferric carboxymaltose and US$ 891 for iron sucrose). Treatment with iron sucrose thus costs US$ 238 more than ferric carboxymaltose, but with a 12% lower chance of hitting the target increase in Hb.

#### Cohort studies

In a non-randomised extension study in patients with kidney disease [47] where treatment duration (up to 43 weeks) depended on the degree of anaemia, IV ferric carboxymaltose resulted in a mean Hb increase of 19 g/L and clinical success (Hb ≥100 g/L, ferritin 100-800 μg/L, TSAT 30-50%) of 72/140 (51%). Most patients had serum ferritin in the range 100-800 μg/L (99%), and TSAT 30-50% (76%).

An open cohort study in patients receiving haemodialysis lasting 10 weeks, with 200 mg IV ferric carboxymaltose two or three times a week during haemodialysis sessions [48] produced a mean increase in Hb of 10 g/L, with 73% and 82% of patients with more than 10 g/L at 2 and 4 weeks. The mean ferritin increase was 403 μg/L, and TSAT increase 16%.

In anaemia secondary to a gastrointestinal disorder, 46 patients were given either 500 mg IV ferric carboxymaltose weekly for four weeks or 1000 mg weekly for two weeks [56]. Most patients had increases in Hb of ≥20 g/L, with 34/46 (74%) having normal Hb (≥140 g/L for men and ≥120 g/L for women). The mean Hb increase was above 20 g/L.

### Withdrawals and adverse events - comparison with oral iron

#### 1 Withdrawal

All cause withdrawals, in all randomised studies combined, were slightly less frequent with IV ferric carboxymaltose than with controls (oral iron, IV iron, and IV placebo; Table 4), though only by about 1% (NNT to prevent one withdrawal 93). There was no significant difference in the comparison with oral iron, nor was there any significant difference between IV ferric carboxymaltose and any control for adverse event or lack of efficacy withdrawal.

**Table 4:** Withdrawals and adverse events

		Number of		Percent with			
				
Outcome	Comparator	Trials	Patients	Ferric carboxymaltose	Control	RB or RR 95% CI	NNTp 95% CI
**Withdrawals**							
All cause	All	10	3835	6.4	7.5	0.8 (0.6 to 0.9)	93 (37 to 180)
	Oral iron	6	1898	8.1	9.3	0.8 (0.6 to 1.03)	not calculated
Adverse event	All	8	3319	1.0	1.6	0.6 (0.3 to 1.02)	not calculated
	Oral iron	6	1898	1.5	1.9	0.7 (0.3 to 1.4)	not calculated
Lack of efficacy	All	7	2967	0.6	0.8	0.8 (0.4 to 1.7)	not calculated
	Oral iron	5	1546	1.1	1.2	0.8 (0.4 to 1.9)	not calculated
**Adverse events**							**NNH** **95% CI**
At least 1 AE	All	8	2951	41	38	1.1 (1.0 to 1.2)	not calculated
	Oral iron	5	1539	48	53	1.0 (0.9 to 1.1)	not calculated
Death	All	10	3762	0.53	0.3	1.3 (0.5 to 3.4)	not calculated
	Oral iron	6	1891	0.38	0.0	1.7 (0.4 to 6.6)	not calculated
Serious AE	All	8	3303	2.5	2.3	1.0 (0.6 to 1.5)	not calculated
	Oral iron	6	1891	3.1	2.3	1.3 (0.7 to 2.2)	not calculated
Hypotension	All	6	2694	1.5	1.0	1.5 (0.8 to 2.7)	not calculated
	Oral iron	4	1339	1.3	0.0	4.7 (1.1 to 21)	79 (44 to 390)

#### 2 Adverse events

No significant difference was found between IV ferric carboxymaltose for patients experiencing at least one adverse event, death, and any serious adverse event. For hypotension, IV ferric carboxymaltose produced significantly more events than oral iron, with a NNH of 79 (Table 4). Hypotension associated with IV ferric carboxymaltose was not a factor in any deaths.

#### 3 Adverse events by body systems

Adverse events were reported according to body systems, and Table 5 shows analyses for IV ferric carboxymaltose compared with oral iron and with IV placebo. In the comparison with oral iron, IV ferric carboxymaltose produced fewer gastrointestinal events (13% compared with 32%, NNTp 5), but more general and administrative site events (NNH 15) and abnormalities in metabolism, nutrition, and investigations (NNH 17). Compared with IV placebo, IV ferric carboxymaltose produced more gastrointestinal and general and administrative site events (NNH 34 and 25, respectively).

**Table 5:** Adverse events by body system and specific adverse events

	Number of		Percent with			
			
Outcome	Trials	Patients	Ferric carboxymaltose	Control	RB or RR 95% CI	NNTp/H 95% CI
**Comparison with oral iron**						
**Body system and preferred term**						
GI disorder	5	1539	13	32	0.44 (0.36 to 0.54)	5.4 (4.4 to 7.1)
General, administrative site	5	1539	11	4	2.8 (1.9 to 4.2)	**15 (11 to 24)**
Infection, infestation	5	1539	14	12	1.2 (0.9 to 1.6)	not calculated
Metabolism, nutrition, investigation	4	1195	11	5	2.2 (1.4 to 3.4)	**17 (11 to 33)**
Nervous system	5	1539	10	9	1.3 (0.9 to 1.7)	not calculated
**Specific adverse events**						
Constipation	4	1339	3	13	0.3 (0.2 to 0.4)	9.8 (7.6 to 14)
Diarrhoea	3	906	2	5	0.5 (0.2 to 0.9)	33 (18 to 230)
Nausea/vomiting	3	906	3	10	0.4 (0.2 to 0.6)	14 (9.5 to 27)
Headache	5	1539	7	7	1.2 (0.8 to 1.7)	not calculated

**Comparison with IV saline**						
**Body system and preferred term**						
GI disorder	2	1577	8	5	1.6 (1.1 to 2.4)	**34 (19 to 210)**
General, administrative site	2	1577	6	2	2.5 (1.5 to 4.3)	**25 (17 to 49)**
Infection, infestation	2	1577	9	6	1.1 (0.8 to 1.6)	not calculated
Nervous system	2	1577	8	6	1.2 (0.9 to 1.8)	not calculated
Respiratory system	2	1577	2	2	0.8 (0.4 to 1.5)	not calculated

Note NNTp in normal text, NNH when bold

#### 4 Specific adverse events

Table 5 also shows specific adverse events mentioned in at least three comparisons between IV ferric carboxymaltose and oral iron. IV ferric carboxymaltose produced less constipation (3% vs 13%, NNTp 10), nausea and vomiting (3% vs 10%, NNTp 14), and diarrhoea (2% vs 5%, NNTp 33).

### Adverse events - active comparisons and cohort studies

In the randomised comparison of IV ferric carboxymaltose with IV placebo in patients with heart failure [42], all cause withdrawal occurred in 9% of patients on ferric carboxymaltose and 13% with placebo. Deaths occurred less frequently with ferric carboxymaltose (5/304, 1.6%) than with placebo (4/155, 2.6%). The randomised comparison of IV ferric carboxymaltose with IV ferric sucrose in inflammatory bowel disease [43] led to few all cause withdrawals (9% vs 11%), and few patients reported any adverse event (14% vs 11%). One serious adverse event was reported with ferric carboxymaltose, and no deaths in this young population (median age 39 years).

In the three cohort studies [47,48,56] 345 patients were treated with IV ferric carboxymaltose. Of these 75 (21%) withdrew for any reason, and 14 (4%) because of adverse events. At least one adverse event was experienced by 197 (56%), serious adverse events by 35 (10%), and hypotension by 10 (3%).

### Death - all studies

There were 20 deaths. These occurred in 15 patients treated with ferric carboxymaltose because of unstated cardiovascular causes (4), myocardial infarction after withdrawal, cardiac arrest, peripartum cardiomyopathy, acute heart failure, prostate cancer, trauma, perforation following diverticulitis, gastrointestinal bleeding following laparoscopy, pulmonary tuberculosis, infection with Aeromonas pulmoniae, and one unknown cause. With iron sucrose there was one death from cardiac failure, and for placebo there were four deaths with unstated cardiovascular causes. Crude mortality rates were 0.6% (15/2696) with IV ferric carboxymaltose, 0.3% (1/382) with IV iron sucrose, 0.5% (4/762) with placebo, and 0% (0/832) with oral iron.

## Discussion

Our knowledge of iron metabolism is still in a state of rapid development [62-64], but however involved it may turn out, we have to interpret the evidence from completed studies on the basis of the tests used at the time. In the case of the ferric carboxymaltose studies, this meant mainly Hb, serum ferritin, and TSAT. Complications arise when assessing the evidence on anaemia in different conditions and with different causes (chronic kidney disease, uterine or postpartum bleeding, gastrointestinal disease, heart failure), using different comparators (oral iron, IV iron, placebo), and from patients receiving erythropoietin. Moreover, haematological outcomes are reported in different ways, typically average change in value, or response or nonresponse, but with differing definitions of response. Trials also had different regimens for giving IV iron, mainly because of different clinical requirements.

Unfortunately some randomised studies were not blind, and others were non-comparative, which does weaken the strength of the evidence available. Set against this were the relatively large numbers of patients reported on, often concerning appropriate duration, and the consistency of response not only between different trials for similar outcomes, but also between randomised and non-comparative studies. A further strength was the availability of CTRs as well as published articles for a number of trials. CTRs are much more detailed than published reports because they are not constrained by word limits, or limits on tables and figures. Most CTRs, for example, comprised over 100 pages, and at least one over 1,000 pages of details. CTRs have been used for a number of meta-analyses where more insightful examination of clinical trials has been needed. They have been used, for example, in reporting adverse events [33], and determining different outcomes in erectile dysfunction [32] and pain [65].

Comparing IV ferric carboxymaltose with oral iron showed a similar time course for Hb and TSAT, though at each time point results for oral iron were numerically worse. Oral iron had no effect on ferritin. Where response was defined as achievement of target Hb, preset Hb rise, or preset Hb rise together with preset changes in ferritin and/or TSAT, there was a statistically significantly better result for ferric carboxymaltose, with NNTs in the range of 6-7, but as low as 1.5 when clinical success was defined with ferritin changes. The much lower, better, NNT reflected the lack of any effect of oral iron on serum ferritin. However response was defined, the response rate with ferric carboxymaltose was in the range of 70-80% (Table 2).

Ferric carboxymaltose produced significantly greater improvements in mean Hb, ferritin, and TSAT in the five trials that compared them, with an average improvement of about 5 g/L, 163 μg/L, and 5% respectively (Figure 6). These figures were obtained at the end of the trials when the increase in ferritin seen soon after therapy started had declined (Figure 2).

In general, and with large numbers of patients, there was little difference in terms of withdrawals and adverse events, including serious adverse events or hypotension, between ferric carboxymaltose and all comparators or oral iron alone.

There were more deaths in the trial arms using ferric carboxymaltose than with control; they were no more frequent than with placebo or iron sucrose, at between 0.3% and 0.6%, though none occurred with oral iron. In the largest and longest study compared with placebo, death rates were similar in the two groups, and 5 of the 11 deaths with ferric carboxymaltose occurred in that trial [42]. Recent reanalyses of trials of ESAs points to rapid Hb increases as being associated with higher mortality risk [66]. If that finding were replicated with IV iron, with or without use of ESA, it might point to a mechanism where too rapid restoration of iron stores was also associated with an increased mortality risk. The emerging view is that serious adverse events have declined with newer IV iron preparations, such that benefits outweigh any risks [67].

In terms of specific adverse events, ferric carboxymaltose was associated with lower rates of constipation, diarrhoea and nausea or vomiting than oral iron, but injection site reactions were higher compared to other parenteral iron preparations.

It is worth mentioning several points that this analysis was not able to assess. For example, no studies compared ferric carboxymaltose with blood transfusion, and direct comparisons between different IV iron products were limited in number, but clearly of interest. This limited the comparison mainly to that between IV ferric carboxymaltose and oral iron. The rate of decline in Hb after stopping treatment with ferric carboxymaltose or oral iron would have been of interest, particularly in some patient groups. This review goes some way to improving the level of evidence available to support recommendations given for IV iron treatment [38], but is not yet a complete response because of the lack of clinical trials. The review was also limited in being able to obtain useful health economic data except from one recent trial [43] that demonstrated a lower cost for IV ferric carboxymaltose over IV iron sucrose, but with a higher success rate. Recent analyses also show that not treating anaemia in CKD with IV iron or ESAs led to greater mortality and cost [68].

## Conclusions

There is substantial evidence that IV ferric carboxymaltose is effective in treating iron deficiency anaemia in many chronic conditions. The review increases the evidence available to support recommendations given for IV iron treatment, but there are limited trial data comparing different IV iron preparations.

## List of abbreviations

CI: confidence interval; CKD: chronic kidney disease; CTR: clinical trial report; EPO: erythropoietin; ESA: erythropoiesis-stimulating agents; Hb: haemoglobin; IV: intravenous; NCT: National Clinical Trials Identifier; NNH: number needed to harm; NNT: number needed to treat; NNTp: number needed to treat to prevent; RB: relative benefit; RR: relative risk; TSAT: transferrin saturation; WHO: World Health Organisation.

## Competing interests

The authors declare that they have no competing interests.

## Authors' contributions

RAM and HG were involved with the original concept, planning the study, writing it, analysis, and preparing a manuscript; PR and JA read the paper as it developed and guided its development. All authors read and approved the final manuscript.

## Authors information

RAM - MA DPhil CChem FRSC FRCA DSc - has been involved with systematic reviews and meta-analyses, and method development in pain and other areas

HG - BM BCh DPhil MRCGP - works in secondary care and evidence-based medicine

PR - FRCP FRCPath - has been involved in prospective clinical trials, systematic reviews, meta-analysis and production of clinical guidelines in Haemostasis and Thrombosis.

JA - MRPharms DipClinPharm - specialising in haematology.

## Pre-publication history

The pre-publication history for this paper can be accessed here:

http://www.biomedcentral.com/1471-2326/11/4/prepub

## Supplementary Material

Additional file 1 **Flowchart of searches**. Click here for file

Additional file 2 **Details of individual studies**. Click here for file
