# Supplementary figures and images for: Meta-analysis of efficacy and safety of intravenous ferric carboxymaltose (Ferinject) from clinical trial reports and published trial data
Source: BMC Blood Disord. 2011 Sep 24;11:4. doi: 10.1186/1471-2326-11-4 (PMC3206450; doi:10.1186/1471-2326-11-4)

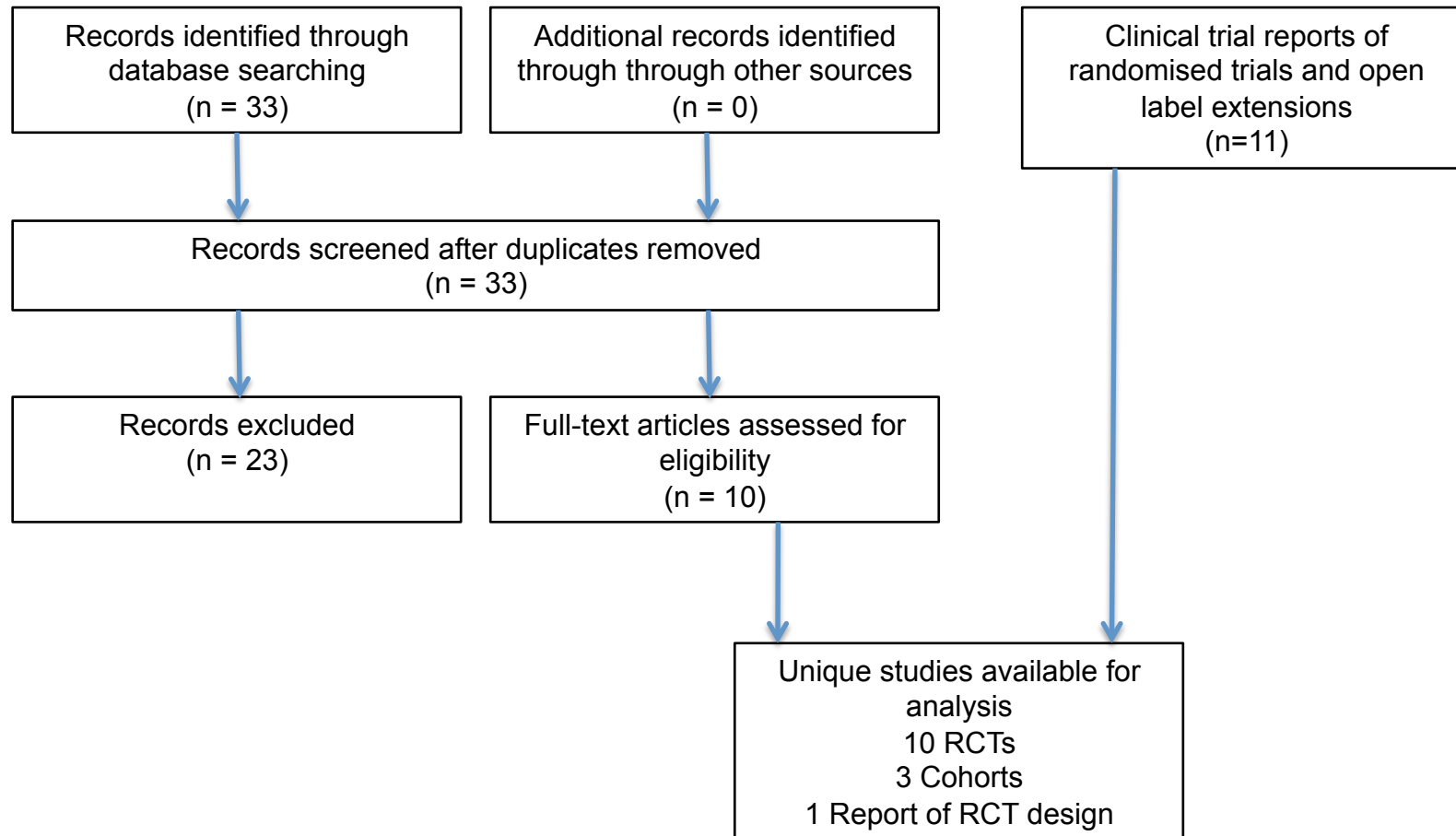

Supplement: Additional file 1 — Flowchart of searches. [file 1471-2326-11-4-S1.PDF]
