# Supplementary material for: Meta-analysis of efficacy and safety of intravenous ferric carboxymaltose (Ferinject) from clinical trial reports and published trial data
Source: BMC Blood Disord. 2011 Sep 24;11:4. doi: 10.1186/1471-2326-11-4 (PMC3206450; doi:10.1186/1471-2326-11-4)
Supplement: Additional file 2 — Details of individual studies. [file 1471-2326-11-4-S2.PDF]

Study	Trial characteristics	Inclusion criteria	Exclusion criteria	Dosing schedules	ITT definition<br><br>Numbers in trial	Measurements	Duration	Patient characteristics	Oxford quality score	Withdrawals	Efficacy<br>Hb	Efficacy<br>ferritin, TSAT, and reticulocytes	Adverse events
**Kidney disease**													
1VIT04004 [44]<br>Qunibi et al. Nephrol Dial Transplant 2010 doi: 10.1093/ndt/gfq613 [45]	Open-label, multicenter, randomized, active-control, parallel groupstudy<br>Patients with non-dialysis dependent CKD who required iron supplementation<br>Stratification by degree of renal function impairment and baseline Hb level	Criteria for randomisation included a hemoglobin ≤ 11.0 g/dL (on two occasions within a week), TSAT ≤ 25%, ferritin ≤ 300 ng/mL, a fixed dose of EPO x 8 weeks (a dose of 0 was permitted) and no parenteral iron for 12 weeks<br>GFR ≤ 45 ml/min/1.73 sqm<br>Age ≥12 years, male or female<br>EPO remained unchanged after randomisation	Hypersensitivity to ferrous sulphate or FCM<br>Other types of anaemia or untreated B12 or folate deficiency<br>Fe storage disorder<br>Recent parenteral iron or blood transfusion, or recent significant blood loss<br>History of gastrointestinal problems with oral iron<br>Current treatment for bronchospasm<br>Use of myelosuppressive medicines<br>Other serious chronic illness and/or recent exacerbation or acute illness or surgery within one month of randomisation<br>Recent parenteral iron or blood transfusion, or recent significant blood loss	1 FCM 1,000 mg IV over 15 minutes day 0, with 500 mg at day 17 and/or day 31 if needed (at baseline maximum dose was 15 mg/kg if weight below 66 kg)<br>2 Ferrous sulfate as 325 mg tablets (65 mg elemental iron) orally three times daily with 8 ounces of tap water, 1 hour before meals.<br><br>Total dose of iron received:<br>FCM = 1218 ± 333 mg (median 1000 mg)<br>Oral iron = 9332 ± 2638 mg (median 10338 mg)	ITT defined as patients who<br>• received at least 1 dose of randomized study medication,<br>• had stable EPO for at least 8 weeks before randomization,<br>• had at least 1 post-baseline hemoglobin assessment, and<br>• had NDD-CKD characterized by a GFR ≤ 45 mL/min/1.73 sqm<br><br>FCM = 152 randomised, 144 ITT<br>Oral iron = 103 randomised, 101 ITT	Hb increase ≥10 g/L<br>Hb increase ≥10 g/L plus ferritin increase ≥160 μg/L<br>Various combinations, in EPO and non-EPO patients, and adverse events	Treatment phase 8 weeks, with maximum 27 weeks<br>Visits on days 0, 14, 28, 43, and 56 after randomisation	Age - median 67 years (range 25-89)<br>Women - 67%<br>Caucasian - 53%<br>Black - 27%<br>Other - 20%<br>All had concomitant medical conditions, and all received concomitant medications<br><br>Baseline<br>Hb - median 103 g/L (range 76-111); 39% ≤100<br>Ferritin - median 90 μg/L (range 2-301); 52% <100<br>TSAT - median 15.5% (range 3.5-25), 76% ≤20%	Randomisation = 2<br>Double blind = 0<br>Withdrawals = 1<br>Total = 3/5	All cause<br>FCM = 13/152<br>Oral iron = 19/103<br><br>Adverse event<br>FCM = 4/152<br>Oral iron = 3/103<br><br>Lack of efficacy (EPO, transfusion)<br>FCM = 6/152<br>Oral iron = 7/103	At some time during study period<br>Hb increase ≥10 g/L at some time<br>FCM = 87/144<br>Oral iron = 35/101<br><br>Hb increase ≥10 g/L plus ferritin increase ≥160 μg/L<br>FCM = 87/144<br>Oral iron = 0/101<br><br>Mean Hb change from baseline to day 56/end of study<br>FCM = 10 ± 11 g/L<br>Oral iron = 7 ± 13 g/L	Ferritin - mean increase by day 56<br>FCM = 359 ± 178 μg/L<br>Oral iron = 26 ± 49 μg/L<br><br>TSAT - mean increase by day 56<br>FCM = 12 ± 8.8%<br>Oral iron = 7.0 ±10.3%<br><br>Reticulocytes - mean change to day 56<br>FCM = -0.1 ± 0.7 %<br>Oral iron = 0.01 ± 0.06%<br>(NB all ≥2 weeks after iv dosing)	At least 1 adverse event<br>FCM = 64/147<br>Oral iron = 61/103<br><br>Death<br>FCM = 2 (prostate cancer, trauma)<br>Oral iron = 0<br><br>Serious adverse events<br>FCM = 13/147<br>Oral iron = 10/103<br>(none considered drug related)<br><br>Hypotension<br>FCM = 5/147<br>Oral iron = 0/103<br>(NB all ≥2 weeks after IV dosing)
1VIT04005 [46]	Open, non randomised extension study of VIT04004	Patient completing VIT04004, and those discontinuing because of use or change in use of EPO, or nonprotocol use of iron	As VIT04004 except for treatment with trial drug	Dosing schedule depending on previous scheduled visit results<br><br>For TSAT ≥30% and ferritin ≥500 μg/L, no FCM<br>For TSAT <25% and ferritin <300 μg/L, maximum dose of FCM 1,000 mg (15 mg/kg to 66 kg)<br>Others, maximum FCM 500 mg (15 mg/kg to maximum 500 mg) administed within one week	145 enrolled<br>127 at least 1 dose FCM in the extention was efficacy ITT<br>140 with FCM in either trial formed safety ITT	Clinical success - Hb ≥110 g/L, TSAT 30-50%, ferritin 100-800 μg/L<br>Sustained success - clinical success on more than 50% of visits	Up to 306 days	Age - median 66 years (range 29-90)<br>Women - 67%<br>Caucasian - 52%<br>Black - 27%<br>Other - 21%<br>All had concomitant medical conditions, and all received concomitant medications<br><br>Baseline<br>Hb - median 105 g/L (range 76-134); 40% ≤100<br>Ferritin - median 99 μg/L (range 6-370); 64% <100<br>TSAT - median 18% (range 3.4-29), 83% ≤20	Not applicable	All cause<br>FCM = 41/145<br><br>Adverse event<br>FCM = 3/145<br><br>Lack of efficacy (EPO, transfusion)<br>FCM = 7/145	Clinical success 72/140<br>Sustained success 14/140<br><br>Mean Hb increase - 19 g/L increase from baseline to highest Hb; 88% ≥110 g/L<br><br>No meaningful changes in reticulocyte count about 1.5%	Mean ferritin increase - 745 μg/L increrase from baseline to highest ferritin; 99% in range 100-800 μg/L<br><br>Mean TSAT increase - 20% increase from baseline to highest TSAT; 76% in range 30-50%	At least 1 adverse event<br>FCM = 84/127<br><br>Death<br>FCM = 2 (prerforation secondary to diverticultis, GI bleed following laparoscopy)<br><br>Serious adverse events<br>FCM = 23/127 (none considered drug related)<br><br>Hypotension<br>FCM = 2/127<br>(both a t ≥14 days post IV dosing)

VIT-IV-CL-015 [47]	Open-label, multicenter, randomized, active-control, parallel groupstudy<br>Patients on dialysis or haemofiltration secondary to chronic renal failure who required iron supplementation<br>Stratification by degree of renal function and Hb level	Either sex, aged 18-80 years, with iron deficiency secondary to chronic renal failure requiring dialysis<br>Hb ≤115 g/L AND serum ferritin <200 μg/L OR TSAT <20%<br>EPO stable for at least 8 weeks if used	Hypersensitivity to either treatment<br>Other types of anaemia or untreated B12 or folate deficiency<br>Recent parenteral iron or blood transfusion, or recent significant blood loss<br>Significant cardiovascular disease, congestive heart failure, or poorly controlled hypertension<br>Recent treatment with investigational drugs	Dosing was 2-3 times weekly at dialysis until individual calculated dose was reached.<br>FCM (50 mg/mL iron) IV directly into haemodialysis venous line 1 hour after start of session<br>Venofer (iron sucrose; 20 mg/mL iron) injected over 10 minutes	240 randomised patients<br>237 received at least one dose of study drug (safety)<br>234 received one dose and provided data (ITT)<br><br>FCM = 119 safety<br>Venofer = 118 safety<br>FCM = 118 ITT<br>Venofer = 116 ITT<br>FCM = 97 PP<br>Venofer = 86 PP	Hb increase ≥10 g/L<br>Clinical success: Hb increase ≥110 g/L (where baseline ≤100 g/L) or ≥120 g/L (where baseline >100 g/L); ferritin 200-800 μg/L; TSAT 20-50%	Four weeks	Provided for PP only:<br>Mean age 52 years (range 22-80)<br>Women - 43%<br>Caucasian - 98%<br>99% had other medical conditions<br>39% on EPO<br>Mean iron deficit 1390 mg<br><br>Hv ≤100 g/L - 68%	Randomisation = 2<br>Double blind = 0<br>Withdrawals = 1<br>Total = 3/5	All cause<br>FCM = 9/119<br>Venofer = 16/118<br><br>Adverse event<br>FCM = 2/119<br>Venofer = 7/118<br><br>Lack of efficacy (EPO, transfusion)<br>FCM = 0/119<br>Venofer = 0/118	Increase of ≥10 g/L at 4 weeks<br>FCM = 52/118<br>Venofer = 41/116<br><br>Mean maximum Hb increase<br>FCM = 14 g/L<br>Venofer = 11 g/L<br><br>Clinical success (Hb ≥110/120 g/L, PP)<br>FCM = 24/97<br>Venofer = 9/86	Maximum ferritin increase<br>FCM = 714 μg/L<br>Venofer = 520 μg/L<br><br>Ferritin 200-800 μg/L at week 4<br>FCM = 64/97<br>Venofer = 65/86<br><br>Maximum TSAT increase<br>FCM = 32%<br>Venofer = 20%<br><br>TSAT 20-50% at week 4<br>FCM = 64/97<br>Venofer = 56/86	At least 1 adverse event<br>FCM = 51/119<br>Venofer = 47/118<br><br>Death<br>FCM = 1/119 (AMI, after withdrawal)<br>Venofer = 0/118<br><br>Serious adverse events<br>FCM = 6/119<br>Venofer = 8/118<br><br>Hypotension<br>FCM = 12/119<br>Venofer = 12/118
53214 [48]<br>Covic & Mircescu. Nephrol Dial Transplant 2010 25: 2722-2730 [49]	Open, non randomised, non comparative study<br>Patients having maintenance haemodialysis	Either sex, aged 18-65 years, having 2 or 3 haelmodialysis sessions a week<br>Clinically stable<br>Hb ≤110 g/L AND serum ferritin <200 μg/L OR TSAT <20%<br>No new EPO within one month, and/or stable dosing	Hypersensitivity<br>Other types of anaemia or untreated B12 or folate deficiency<br>Recent parenteral iron or blood transfusion, or recent significant blood loss<br>Recent treatment with investigational drug	Patients received 200 mg FCM 2 or 3 times per week during each haemodialysis session<br>Maximum dose 2,400 mg<br>Target Hb 150 g/L	162 patients enrolled and received drug, with 150 completing	Various haematological variables	10 weeks	Mean age 45 years (range 18-65)<br>Women - 44%<br>Caucasian - 69%<br>Black/mixed - 28%	Not applicable	17 all cause discontinuations<br>5 AE discontinuations<br>2 Lack of efficacy (additional iron preparations)	Mean final Hb increase 10 g/L<br><br>73% and 82% Hb increase of ≥10 g/L 2 and 4 weeks after final treatment	Mean final 2 weeks after final treatment ferritin increase 403 μg/L<br><br>Mean final 2 weeks after final treatment TSAT increase 16%	At least 1 AE 89/162<br>Death 2/162 (pulmonary tuberculosis, acute heart failure)<br>Serious AE 12/162<br>Hypotension 8/162
**Postpartum anaemia and heavy uterine bleeding**													
VIT-IV-CL-009 [50]<br>Breymann et al. Int J Gynecol Obstet 2008 101: 67-73 [51]	Open-label, multicenter, randomized, active-control, parallel groupstudy<br>Women with postpartum anaemia<br>Stratification by country and level of anaemia	Women aged ≥18 years with postpartum anaemia within 6 days of delivery<br>Hb ≤105 g/L (average of two samples of separate days)	Transfusion, EPO, or parenteral iron within 30 days<br>Hypersensitivity to therapies<br>Other types of anaemia or untreated B12 or folate deficiency<br>Iron storage disorder<br>Other serious illness	FCM given as IV infusion to maximum of 1,000 mg iron per infusion<br>Infusions on Day 1, 8 and 15 to achieve individual iron requirement<br>Oral ferrous sulphate 100 mg minimum of one hour before food, twice daily for 12 weeks<br><br>Total dose of iron received:<br>FCM = 1347 (range 600-2200 mg)	349 patients randomised, and 344 received at least one dose of study drug (ITT definition)<br><br>FCM = 227<br>Oral iron = 117<br><br>Some data reported as per protocol, n = 268	Hb, ferritin, and TSAT and change over time<br>Target values were Hb - 120-160 g/L; ferritin - 50-800 μg/L; TSAT - 20-50%<br>Transfusion requirement	Up to 12 weeks	Age - mean 28 years (range 18-44)<br>Caucasian - 99%<br>Gestational age 39 weeks<br><br>Baseline<br>Hb - mean 96 g/L (range 52-145)<br>Ferritin - mean 38 μg/L (range 0-605)<br>TSAT - mean 12% (range 2-54)<br><br>Individual iron deficit<br>FCM = 1,370 ± 182 mg<br>Oral iron = 1,384 ± 186 mg	Randomisation = 2<br>Double blind = 0<br>Withdrawals = 1<br>Total = 3/5	All cause<br>FCM = 29/227<br>Oral iron = 15/117<br><br>Adverse event<br>FCM = 3/227<br>Oral iron = 1/117<br><br>Lack of efficacy (EPO, transfusion)<br>FCM = 0/227<br>Oral iron = 0/117	Hb responders (week 12)<br>FCM = 152/179<br>Oral iron = 73/89<br><br>Mean Hb change (week 12)<br>FCM = 34 ± 18 g/L<br>Oral iron = 33 ± 17 g/L	Ferritin responders (week 12)<br>FCM = 139/179<br>Oral iron = 29/89<br><br>TSAT responders (week 12)<br>FCM = 139/179<br>Oral iron = 59/89<br><br>Mean ferritin change (week 12)<br>FCM = 124 ± 126 μg/L<br>Oral iron = 11 ± 39 μg/L<br><br>Mean TSAT change (week 12)<br>FCM = 23 ±17%<br>Oral iron = 14 ±14%	At least 1 adverse event<br>FCM = 59/227<br>Oral iron = 26/117<br><br>Death<br>FCM = 0/227<br>Oral iron = 0/117<br><br>Serious adverse events<br>FCM = 2/227<br>Oral iron = 0/117<br><br>Hypotension<br>FCM = 1/227<br>Oral iron = 0/117<br>(1 hour after first IV dose)
1VIT06011 [52]<br>Seid et al. Am J Obstet Gynecol 2008 199:435.e1-435.e7 [53]	Open-label, multicenter, randomized, active-control, parallel group study<br>Women with postpartum anaemia<br>Stratification level of anaemia (Hb and ferritin)	Women with postpartum anaemia within 10 days of delivery<br>Hb ≤100 g/L (average of two samples)<br>Ferritin <100 μg/L	Ferritin >100 μg/L<br>GI problems with iron products<br>Hypersensitivity to therapies<br>Other types of anaemia or untreated B12 or folate deficiency<br>Significant recent vaginal bleeding<br>Other serious illness	FCM given as slow IV injection where iron dose ≤500 mg, and slow IV injection or infusion over 15 minutes where dose was 600-1000 mg. Dosing was based on calculated iron deficit, and continued at weekly intervals<br>Oral ferrous sulphate 325 mg three times a day for 6 weeks<br><br>Total dose of iron received:<br>FCM = 1503 ± 384 mg (median 1500 mg)<br>Oral iron = 7906 ± 981 mg (median 8190 mg)	The ITT population was defined as all randomised subjects who had postpartum anemia characterised by an average of 2 baseline central laboratory Hb values <110 g/L.<br><br>FCM = 143<br>Oral iron = 148	Hb increase ≥30 g/L at any time<br>Sustained success - Hb of >120 g/L at end of study<br>Change in Hb, ferritin, TSAT	Six weeks	Age - mean 26 years (range 16-43)<br>Caucasian - 69%<br>Blood loss on delivery 770 mL (range 600-1500 mL)<br><br>Baseline<br>Hb - mean 89 g/L (range 57-104)<br>Ferritin - mean 24 μg/L (range 4-124)<br>TSAT - mean 9% (range 2-28)	Randomisation = 2<br>Double blind = 0<br>Withdrawals = 1<br>Total = 3/5	All cause<br>FCM = 4/143<br>Oral iron = 3/148<br><br>Adverse event<br>FCM = 0/143<br>Oral iron = 0/148<br><br>Lack of efficacy (EPO, transfusion)<br>FCM = 1/143<br>Oral iron = 1/148	Hb responders (≥120 g/L at any time)<br>FCM = 127/143<br>Oral iron = 98/148<br><br>Increase in Hb ≥30 g/L at any time<br>FCM = 127/139<br>Oral iron = 95/147<br><br>Sustained success<br>FCM = 117/137<br>Oral iron = 83/143<br><br>Success plus ferritin change ≥160 μg/L any time<br>FCM = 127/139<br>Oral iron = 0/147<br><br>Mean Hb change week 6<br>FCM = 40 ± 11 g/L	Mean ferritin change (week 6)<br>FCM = 226 ± 118 μg/L<br>Oral iron = 3 ± 20 μg/L<br><br>Mean TSAT change (week 6)<br>FCM = 29 ±12%<br>Oral iron = 17 ±18%	At least 1 adverse event<br>FCM = 65/142<br>Oral iron = 83/147<br><br>Death<br>FCM = 0/142<br>Oral iron = 0/147<br><br>Serious adverse events<br>FCM = 4/142<br>Oral iron = 4/147<br><br>Hypotension<br>FCM = 1/142<br>Oral iron = 0/147

1VIT04002<br>1VIT04003 [54]<br>Van Wyck et al. Transfusion 2009 49: 2719-2728 [41]	Open-label, multicenter, randomized, active-control, parallel groupstudy<br>Women with anaemia due to heavy uterine bleeding<br>Stratification level of anaemia and severity of uterine blood loss	Women aged ≥18 years<br>Hb - average of two samples below 110 g/L, ferritin ≤100 μg/L, TSAT ≤25%<br>Heavy uterine bleeding for 6 months:<br>• Inability to control with tampons alone<br>• Excessive use of pads or tampons<br>• Passage of clots<br>• Period duration >7 days	Hypersensitivity to therapies<br>Blood transfusion or parenteral iron within 8 weeks<br>EPO within 8 weeks or during study<br>Other types of anaemia or untreated B12 or folate deficiency<br>Fe storage disorder<br>Medication likely to affect vaginal bleeding, or insertion intrauterine contraceptive device, within 8-12 weeks<br>Significant recent vaginal bleeding<br>Other serious illness<br>Current treatment for asthma	FCM given as slow IV undiluted injection *push* where iron dose ≤200 mg, and slow IV i infusion over 6 or 15 minutes where dose was 300-1000 mg. Dosing was based on calculated iron deficit, and continued at weekly intervals<br>Oral ferrous sulphate 325 mg three times a day for 6 weeks<br><br>Total dose of iron received:<br>FCM = 1568 ± 422 mg (median 1500)<br>Oral iron = 7302 ± 1937 mg (median 7995)	477 women randomised<br>456 randomised and received at least one dose<br><br>FCM = 230<br>Oral iron = 225<br><br>Completers (per protocol)<br>FCM = 211<br>Oral iron = 212	Success defined as increase of Hb from baseline of 20 g/L at any time<br>Change in Hb, ferritin, TSAT<br>Combinations of success based on change in Hb, ferritin, and TSAT	Six weeks	Age - mean 39 years (range 18-54)<br>Severe or very severe bleeding - 58%<br>Black - 48%<br>Caucasian - 27%<br>Hispanic - 22%<br><br>Baseline<br>Hb - mean 94 g/L (range 49-111); 50% <95 g/L<br>Ferritin - mean 7 μg/L (range 1-82)<br>TSAT - mean 6% (range 1-25)	Randomisation = 2<br>Double blind = 0<br>Withdrawals = 1<br>Total = 3/5	All cause<br>FCM = 19/230<br>Oral iron = 14/226<br><br>Adverse event<br>FCM = 2/230<br>Oral iron = 3/226<br><br>Lack of efficacy (EPO, transfusion)<br>FCM = 1/230<br>Oral iron = 0/226	Hb responders (≥120 g/L at any time)<br>FCM = 166/228<br>Oral iron = 112/225<br><br>Hb increase ≥20 g/L at any time<br>FCM = 187/228<br>Oral iron = 139/225<br><br>Increase in Hb ≥30 g/L at any time<br>FCM = 121/228<br>Oral iron = 80/225<br><br>Success plus ferritin change ≥160 μg/L any time<br>FCM = 186/228<br>Oral iron = 0/225<br><br>Mean change in Hb at any time<br>FCM 33 ± 15 g/L<br>Oral iron 26 ± 16 g/L	Mean ferritin change (week 6)<br>FCM = 175 ± 133 μg/L<br>Oral iron = 17 ± 17 μg/L<br><br>Mean TSAT change (week 6)<br>FCM = 19 ± 8%<br>Oral iron = 19 ± 22%<br><br>Reticulocytes - mean change to week 6<br>FCM = -0.2 ± 1.3 %<br>Oral iron = 0.01 ± 1.1%	At least 1 adverse event<br>FCM = 157/230<br>Oral iron = 149/226<br><br>Death<br>FCM = 0/230<br>Oral iron = 0/226<br><br>Serious adverse events<br>FCM = 3/230<br>Oral iron = 3/226<br><br>Hypotension<br>FCM = 0/230<br>Oral iron = 0/226
Van Wyck et al. Obst & Gynecol 2007 110:267-278 [55]	Open-label, multicenter, randomized, active-control, parallel group study<br>Women with postpartum anaemia<br>Stratification level of anaemia (Hb and ferritin)	Women with postpartum anaemia within 10 days of delivery<br>Hb ≤100 g/L	Ferritin >500 μg/L<br>TSAT ≥50%<br>GI problems with iron products<br>Hypersensitivity to therapies<br>Other types of anaemia or untreated B12 or folate deficiency<br>Significant recent vaginal bleeding<br>Other serious illness<br>EPO within 3 months	FCM given to calculated iron deficit at 15 mg/kg, not exceeding 1000 mg a day, with subsequent doses one week later, IV over 1-15 minutes, depending on volume<br>Oral iron 325 mg tablets three times daily (195 mg iron)<br><br>Total dose of iron received:<br>FCM = 1403<br>Oral iron = 6764	ITT (randomised and one dose of medication) = 352<br><br>FCM = 174<br>Oral iron = 178<br><br>Number with dose and measurements<br>FCM = 169<br>Oral iron = 168	Patient with ≥20 g/L increase in Hb<br>Patients with Hb ≥120 g/L<br>Hb increase of ≥20 g/L and ferrinin increase ≥160 μg/L	Six weeks	Age - mean 26 years<br>Caucasian - 83%<br><br>Baseline<br>Hb - mean 90 ± 10 g/L<br>Ferritin - mean 24 ± 30 μg/L<br>TSAT - mean 10 ± 6%	Randomisation = 1<br>Double blind = 0<br>Withdrawals = 1<br>Total = 2/5	All cause<br>FCM = 9/174<br>Oral iron = 16/178<br><br>Adverse event<br>FCM = 2/174<br>Oral iron = 4/178	Hb responders (≥120 g/L at any time)<br>FCM = 153/169<br>Oral iron = 115/168<br><br>Increase in Hb ≥20 g/L at any time<br>FCM = 163/169<br>Oral iron = 158/168<br><br>Mean Hb change week 6<br>FCM = 44 g/L<br>Oral iron = 33 g/L	Mean ferritin change (week 6)<br>FCM = 210 μg/L<br>Oral iron = 10 μg/L<br><br>Mean TSAT change (week 6)<br>FCM = 29%<br>Oral iron = 14%	Death<br>FCM = 1/174 (peripartum cardiomyopathy)<br>Oral iron = 0/178<br><br>Serious adverse events<br>FCM = 1/174<br>Oral iron = 1/178

**Gastrointestinal cause of anaemia**													
VIT-IV-CL-03 [56]	Open, uncontrolled cohort study<br>Patients with Hb ≤110 g/L with stable disease	All cause<br>C1 - 6/20<br>C2 - 7/26<br><br>Adverse event or intercurrent illness<br>C1 - 4/20<br>C2 - 2/26	Hypersensitivity to therapies<br>Blood transfusion or iv iron within 4weeks<br>Serum ferritin >500 μg/L and serum TfS >45%.<br>Other types of anaemia or untreated B12 or folate deficiency<br>Iron storage disorder<br>Treatment with investigational drug within 4 weeks<br>Other serious illness	Cohort 1: 500 mg (last dose lower depending on Fe requirement) as IV infusion weekly for up to 4 weeks<br>Cohort 2: 1000 mg (last dose lower depending on Fe requirement) as IV infusion weekly for up to 2 weeks<br><br>as determined by total iron requirement	46 patients in the two cohorts		Treatment phase<br>4 or 2 weeks<br>Follow up 2 and 4 weeks after last dose	Age - mean 45 years (range 20-61)<br>Women - 78%<br>Caucasian - 100%<br><br>Baseline<br>Hb - mean 87 g/L<br>Ferritin - mean 4 μg/L<br>TSAT - mean 23%	Not applicable.<br>Cohort 2 begun only when cohort 1 completed	All cause<br>C1 - 6/20<br>C2 - 7/26<br><br>Adverse event or intercurrent illness<br>C1 - 4/20<br>C2 - 2/26	Increase of ≥20 g/L<br>C1 - 15 at 4 week follow up<br>C2 - 18 aT 4 week follow up<br><br>Mean Hb change<br>C1 - 39 g/L (week 4)<br>C2 - 17 g/L (week 2)<br><br>Post treatment - 4 weeks after last dose<br>C1 - 120 g/L<br>C2 - 121 g/L<br><br>"Normal" Hb (≥140 g/L men, ≥120 g/L women)<br>C1 - 15/20<br>C2 - 19/26	Mean ferritin change<br>C1 = 144 μg/L (week 4)<br>C2 = 401 μg/L (week 2)<br><br>Mean ferritin change (4 week follow up)<br>C1 = 57 μg/L (week 4)<br>C2 = 95 μg/L (week 2)	At least 1 adverse event<br>C1 - 11/20<br>C2 - 13/26<br><br>Deaths<br>C1 - 0/20<br>C2 - 0/26<br><br>Serious AE<br>C1 - 0/20<br>C2 - 0/26<br><br>No reports of hypotension
VIT-IV-CL-008 [57]<br>Kulnigg et al. Am J Gastroeneterol 2008 24:1507-1523 [58]	Open-label, multicenter, randomized, active-control, parallel groupstudy<br>Patients with iron deficiency anaemia secondary to chronic inflammatory bowel disease<br>Stratification by sex and country	Adults aged 18-80 years<br>Anaemia secondary to inflammatory bowel disease (Crohn's disease or ulcerative colitis)<br>Hb ≤110 g/l (mean of two values on different days), AND serum ferritin <100 μg/L, OR TSAT <20%<br>iron requirement at least 1,000 mg	Hypersensitivity to therapies<br>Blood transfusion within 4 weeks, EPO within 8 weeks<br>Other types of anaemia or untreated B12 or folate deficiency<br>Treatment with investigational drug within 4 weeks<br>Iron storage disorder<br>Other serious illness	FCM was given as an IV infusion to deliver a maximum of 1,000 mg iron per infusion. Infusions were given on day 1 and weekly until individual iron requirement reached or maximum 3 doses given.<br>Oral ferrous sulphate(Fe 100 mg) capsules were taken twice daily (daily dose 200 mg iron)for 12 weeks	200 randomised and received at least one dose (safety analysis)<br>196 provided efficacy data (ITT population)<br>160 in per protocol population	Hb change from baseline to week 12<br>Number achievinbg target levels of Hb (135-180 g/L men, 120-160 g/L women), ferritin (100 800 μg/L), and TSAT (20-50%)<br>Number with Hb increase of >20 g/L	12 weeks	Age - mean 43 years (range 19-78)<br>Women - 61%<br>Caucasian - 99%<br>Mean iron deficiency 1,448 mg (range 937-2102 mg)<br><br>Baseline<br>Hb - mean 87 g/L (range 50-115 g/L)<br>Ferritin - mean 16 μg/L (range 1-383 μg/L)<br>TSAT - mean 8% (range 1-64%)	Randomisation = 2<br>Double blind = 0<br>Withdrawals = 1<br>Total = 3/5	All cause<br>FCM = 12/137<br>Oral iron = 11/63<br><br>Adverse event<br>FCM = 3/137<br>Oral iron = 4/63<br><br>Lack of efficacy (EPO, transfusion)<br>FCM = 2/137<br>Oral iron = 0/63	PP data<br><br>Mean Hb change (week 12)<br>FCM = 38 ± 20 g/L (111)<br>Oral iron = 38 ± 20 g/L (49)<br><br>Normal Hb week 12 (135-180 men, 120-160 women)<br>FCM = 57/111<br>Oral iron = 23/49<br><br>HB increase ≥20 g/L (week 12)<br>FCM = 90/111<br>Oral iron = 40/49	PP data<br><br>Mean ferritin change (week 12)<br>FCM = 72 ± 100 μg/L<br>Oral iron = 20 ± 60 μg/L<br><br>Ferritin 100-800 μg/L (week 12)<br>FCM = 32/111<br>Oral iron = 2/49<br><br>Mean TSAT change (week 12)<br>FCM = 18 ± 18%<br>Oral iron = 21 ± 25%<br><br>TSAT 20-50% (week 12)<br>FCM = 48/111<br>Oral iron = 23/49	At least 1 adverse event<br>FCM = 78/137<br>Oral iron = 27/63<br><br>Deaths<br>FCM = 1/137 (cardiac arrest)<br>Oral iron = 0/63<br><br>Serious AE<br>FCM = 9/137<br>Oral iron = 0/63<br><br>No reports of hypotension
Estiatiev et al. Gastroenterology 2011, Epub June 12 [43]	Randomised, open comparison between IV FCM with IV iron sucrose (Venofer) with outcomes measured after 12 weeks randomisation by computer generated code	Adults ≥18 years with iron deficiency anaemia (Hb 70-120 g/L {women} or 70-130 g/L {men}) and with mild to moderate inflammatory bowed disease (Chrohn;s disease or ulcerative colitis), and niormal levels of vitamin B12 and folic acid	Patients with IV or oral iron treatment in preceding 4 weeks, or EPO treatment.<br>Other exclusions were chronic alcohol abuse, liver disease, or increased transaminases, surgery with blood loss, plus other sensible exclusions	FCM (1000 mg or 500 mg iron, depending on weight) given as IV infusion to maximum of 1,000 mg iron per infusion<br>Infusions on Day 1, and, if needed days 8 and 15 to achieve individual iron requirement<br>Iron sucrose was given in up to 11 infusions of 200 mg iron over 30 minutes, twice weekly	485 randomised and 483 received at least one dose<br><br>FCM 244<br>Iron sucrose 239	Primary endpoint was Hb increase ≥20g/L at 12 weeks<br>Normalisation of Hb (120 or 130 g/L)<br>TSAT 20-50%<br>Ferritin ≥ 100 μg/L<br>SF-36 QoL measures	12 weeks	Age - Median 39 years (range 18-81)<br>Women- 58%<br><br>Baseline<br>Mean Hb - 102 g/L<br>Mean TSAT - 9.3%<br>Mean ferritin - 16.3 μg/L	Randomisation = 2<br>Double blind = 0<br>Withdrawals = 1<br>Total = 3/5	All cause<br>FCM = 22/244<br>IS = 26/239<br><br>Adverse event<br>FCM = 8/244<br>IS = 8/239<br><br>Lack of efficacy<br>FCM = 0/244<br>IS = 7/239	ITT data<br><br>Hb increase ≥20 g/L<br>FCM = 150/240<br>IS = 115/235<br><br>Hb increase ≥20 g/L or normal Hb<br>FCM = 191/240<br>IS = 167/235<br><br>Normal Hb<br>FCM = 166/240<br>IS = 136/235	ITT data<br><br>TSAT 20-50%<br>FCM = 117/240<br>IS = 76/235<br><br>Ferritin ≥100 μg/L<br>FCM = 96/240<br>IS = 60/235	At least 1 adverse event<br>FCM = 34/244<br>IS = 37/239<br><br>Serious AE<br>FCM = 1/244<br>IS = 0/239<br><br>Deaths<br>FCM = 0/244<br>IS = 0/239

**Iron deficiency anaemia of mixed origin**													
1VIT05006 [59]<br>Bailie et al.<br>Hemodialysis International 2010 14: 47-54 [35]	Randomised, double blind crossover comparison of IV FCM with IV placebo over 7 days for AE only<br>Patients with iron deficiency from any cause<br>Randomisation startified by condition and centre	Adults ≥18 years<br>Hb ≤120 g/L, TSAT ≤25%, and ferritin ≤300 μg/L (CKD, IBD), or ≤100 μg/L in other conditions	Hypersensitivity to therapies<br>Previously received FCM<br>Parenteral iron within previous 4 weeks<br>Fe storage disorders<br>Other serious illness<br>Current treatment for brochospasm	Blinded FCM or placebo, maximum 1,000 mg iron, IV over 15 minutes on day 0, with alternate on day 7<br><br>Total dose of iron received:<br>FCM = 944 ± 155 mg (median 1000)<br>Placebo = 0	598 randomised<br>582 randomised and received at least one dose<br>12 patients enrolled in pharmacokinetic study also enrolled in safety population (tthough that was open), making 594<br>559 of these had both doses	No Hb measures	one week in each crossover arm		Randomisation = 2<br>Double blind = 2<br>Withdrawals = 1<br>Total = 5/5	All cause<br>FCM = 14/592<br>Placebo = 12/592<br><br>Adverse event<br>FCM = 1/592<br>Placebo = 2/592<br><br>Lack of efficacy (EPO, transfusion)<br>FCM = 0/592<br>Placebo = 1/592	No data	No data	At least 1 adverse event<br>FCM = 164/559<br>Placebo = 110/559<br><br>Deaths<br>FCM = 1/559 (Aeromonas pneumonia)<br>Placebo = 0/559<br><br>Serious AE<br>FCM = 2/559<br>Placebo = 4/559<br><br>Hypotension<br>FCM = 0/559<br>Placebo = 1/559
CARS 1 [60]	Randomised, double blind, parallel, placebo and active controlled trial<br>Patients with CHF, renal faiure, and inflammatory bowel disease	Adults ≥18 years	None given	IV FCM or Venofer to calkculated iron deficit with weekly 200 mg doses of iron, then 200 mg every 4 weeks, or placebo IV<br><br>Total dose of iron received:<br>FCM = 1103 ± 279 mg (median 1100)<br>Venofer = 1167 ± 315 mg (median 1200)<br>Placebo = 0	72 patients enrolled<br><br>FCM = 30<br>Venofer = 27<br>Placebo = 15	Change in Hb, ferritin, TSAT	12 weeks	Mean age about 70 years<br>Women - about 64%<br>Caucasian - 100%<br><br>Baseline<br>Hb = 123 g/L<br>Ferritin - range of means 17-77 μg/L<br>TSAT - range of means 16-19%	Randomisation = 2<br>Double blind = 2<br>Withdrawals = 1<br>Total = 5/5	All cause<br>FCM = 0/30<br>Venofer = 3/27<br>Placebo = 2/15	Mean Hb change by week 12<br>FCM = 8.0 ± 12 g/L<br>Venofer 9.0 ± 11 g/L<br>Placebo = -3 ± 18 g/L	Mean ferritin change by week 12<br>FCM = 254 ± 145 μg/L<br>Venofer 230 ± 119 μg/L<br>Placebo = 0 ± 82 μg/L<br><br>Mean TSAT change by week 12<br>FCM = 8.7 ± 13%<br>Venofer 8.5 ± 5.6%<br>Placebo = -2.3 ±12%	At least 1 adverse event<br>FCM = 15/30<br>Venofer = 12/27<br>Placebo = 10/15<br><br>Death<br>FCM = 0/30<br>Venofer = 1/27 (cardiac failure)<br>Placebo = 0/15<br><br>Serious AE<br>FCM = 3/30<br>Venofer = 5/27<br>Placebo = 2/15
**Heart failure**													
Anker et al.<br>NEJM 2009 361: 2436-48 [42]	Randomised, multicentre, double blind, parallel group placebo comparison in patients with heart failure and iron deficiency<br>Startified by country	Ambulatory CHF patients<br>NYHA c;ass II or III<br>LVEF≤40% (II) or 45% (III)<br>Ferritin ≤100 μg/L or 100-300 when TSAT ≤20%<br>Hb 95-135 g/L	Anaemia other than iron deficiency<br>Active infections<br>AST >3 ULN<br>History of transfuion, EPO, or parenteral iron in last 3 months<br>Unstable angina<br>Major cardiovascular intervention within 3 months	Dosing required for iron repletion calculated at baseline<br>Intravenous bolus of 200 mg iron (4 mL) weekly until iron replete then saline every 4 weeks ,but active treatment again if Fe status deteriorated or (placebo group) saline at same intervals as active treatment, until week 24<br>If Hb fell to <90 g/L during study, other management of anaemia as decided by investigator	459 patients randomised and drug administered<br><br>FCM = 304<br>Placebo = 155	Patient Global Assessment, and NYHA functional class at week 24<br>Measurements of Hb, ferritin, and TSAT, and week 24 results reported according to initial Hb level	24 weeks	Mean age 67 years<br>Women - 53%<br>Hypertension - 81%<br><br>Baseline Hb - mean 119 g/L<br>Ferritin - mean 55 μg/L<br>TSAT - mean 17%	Randomisation = 2<br>Double blind = 2<br>Withdrawals = 1<br>Total = 5/5	All cause<br>FCM = 26/304<br>Placebo = 20/155	Final Hb Mean Hb at 24 weeks when initial baseline Hb ≤120 g/L<br>FCM = 127 g/L<br>Placebo = 118 g/L<br><br>Final Hb Mean Hb at 24 weeks when initial baseline Hb >120 g/L<br>FCM = 133 g/L<br>Placebo = 132 g/L	Final ferritin when initial Mean ferritin at 24 weeks when baseline Hb ≤120 g/L<br>FCM = 275 μg/L<br>Placebo = 68 μg/L<br><br>Final ferritin when initial Mean ferritin at 24 weeks when baseline Hb >120 g/L<br>FCM = 349 μg/L<br>Placebo = 80 μg/L<br><br>Final TSAT when initial Mean TSAT at 24 weeks when baseline Hb ≤120 g/L<br>FCM = 29%<br>Placebo = 17%<br><br>Final TSAT when initial Mean TSAT at 24 weeks when baseline Hb >120 g/L<br>FCM = 30%<br>Placebo = 22%	Death<br>FCM = 5/304 (4 cardiovascular causes)<br>Placebo = 4/155 (cardiovascular causes)
